# Diagnosis and imaging in COVID-19 induced myositis

**DOI:** 10.1259/bjrcr.20220134

**Published:** 2023-05-15

**Authors:** Zaynab Abid Sohail, Jawad Naqvi, Leanne Gray

**Affiliations:** 1 Royal Lancaster Infirmary, Lancaster, Lancashire, United Kingdom

## Abstract

The case describes how musculoskeletal disease can manifest in coronavirus disease. The most reported symptoms of covid infection are cough, fever and myalgia. Myalgia and myositis are similar conditions in that they both describe muscular pain and fatigue. However, they are distinguishable in that myalgia is usually benign and self-limiting whilst untreated myositis can lead to serious complications often requiring hospital admission. This case report aims to familiarise physicians with atypical presentations of coronavirus disease to prevent delays in diagnosis and treatment. Timely treatment of covid-induced myositis can decrease in-hospital mortality rates and improve patient outcomes.

## Clinical presentation

A 53-year-old female was admitted to the acute medical unit with a four-day history of worsening lower limb pain associated with pyrexia, lower limb weakness and a rash. The patient tested positive for coronavirus disease (covid-19) four days before admission. The patient had two AstraZeneca COVID-19 vaccinations, one in January 2021 and the other in April 2021 followed by a Pfizer booster in October 2021. The Pfizer booster was administered 6 months prior to admission. The patient was not tested for coronavirus antibodies. The patient had no significant previous medical history and was not on any regular medications. She had a ten-pack-year smoking history and no illicit drug use history. Examination revealed livedo reticularis of the lower limbs, non-palpable peripheral pulses, and profound, proximal and symmetric acute weakness of the lower limbs.

### Investigations/ Imaging findings

Initial work-up included blood results shown in Table 1:

**Table 1.** Blood results

Test	Result	Reference Range
D-dimer	444	80–500 mg l^−1^
C-Reactive Protein (CRP)	8.9	<5 mg l^−1^
Erythrocyte Sedimentation Rate (ESR)	24	0–20 mm/hr
Creatine Kinase (CK)	524	25–200 Units/L
White Cell Count (WCC)	14.9	4–10 × 10^9^ l^−1^
Neutrophils	10.6	2–7.5 × 10^9^ l^−1^
Creatinine	56	45–84 µmol l^−1^
Urea	5.7	2.5–7.8 mmol l^−1^
Estimated Glomerular Filtration Rate (eGFR)	>90	>90

The patient had a CT angiogram (CTA) of the legs, excluding acute occlusion in the lower limb arterial tree.

**Figure 1.**
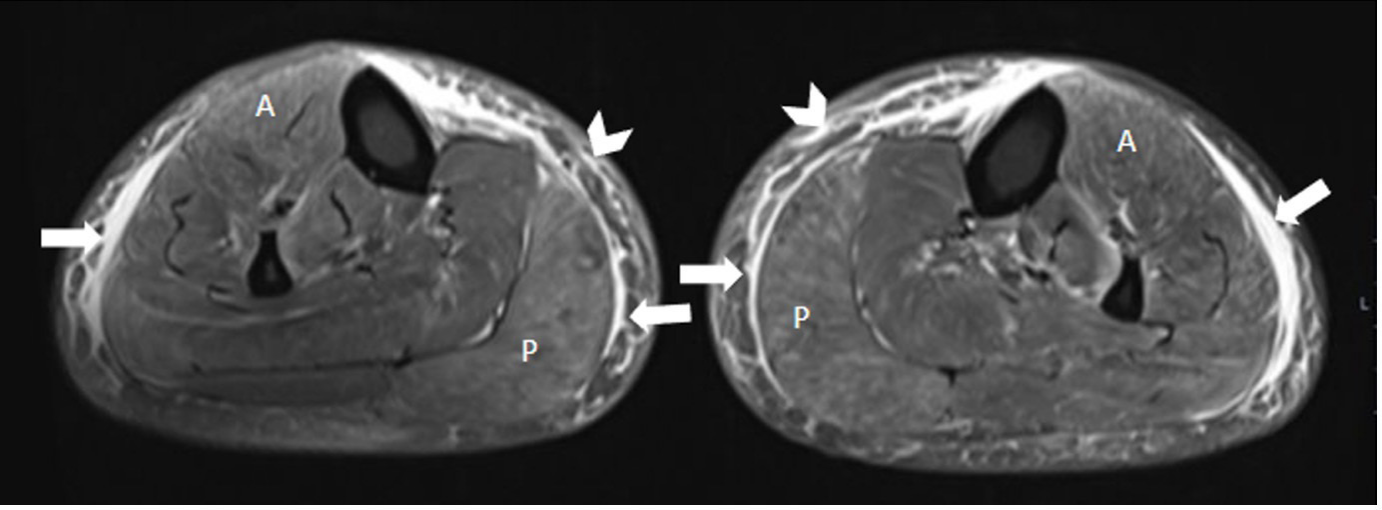
Labelled axial proton density fat-saturated image of bilateral lower legs. Diffuse muscle oedema is seen as hyperintense feathery signal changes within the muscle fibres of all compartments, most notable in the anterior compartment (**A**) and posterior compartments (**P**). Perifascial fluid is also seen (arrows) and subcutaneous oedema (chevron).

Repeat blood results three days post-admission showed an increase in Creatine Kinase (CK) from 524 to 24,250 U l^−1^. To investigate this further, the patient underwent an MRI of the lower legs (Figure 1).

**Figure 2.**
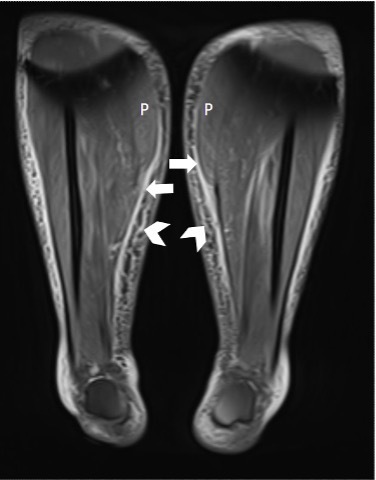
Labelled coronal proton density fat-saturated image of bilateral lower legs demonstrating extent of inflammatory change with feathery oedema seen in the posterior compartment (**P**) affecting the medial gastrocnemius muscles and fascial (white arrows) and subcutaneous adipose oedema (chevrons).

The MRI (Figures 1 and 2) shows predominantly symmetrical diffuse muscle oedema in all the muscle compartments of bilateral lower legs, mostly seen in the right anterior and bilateral posterior compartments. There is associated perifascial fluid as well as subcutaneous adipose tissue oedema.

## Differential diagnosis

The main differentials include idiopathic inflammatory myopathies (IIM) such as polymyositis, dermatomyositis (DM) and inclusion body myositis. Secondary causes of inflammatory myopathy include myositis associated with malignancy, infection, drugs and toxins.^1^ A rise in CK is significantly associated with IIM.^2^ Additional diagnostic tools such as medical history taking, clinical examination and radiological findings can support the diagnosis of IIM.^3^ It can then be confirmed by serological, microbiological and histological results. The presence of a rash could point towards a diagnosis of DM however, the expected characteristics would include a Heliotrope rash, Gottron’s papules and/or Gottron’s sign; linear extensor erythema which appear as purple papules or plaques.^4,5^ The livedoid rash seen in this case could be benign or associated with infection, arteriopathy or underlying vasculitis.^6^

Key criteria to diagnose IIM require that secondary causes of myopathy are first excluded.^5^ In this case, the patient presented with symptoms following infection with covid-19 therefore a diagnosis of myopathy secondary to viral infection is most likely. In infectious myopathy, causal agents can be bacterial, typically caused by staphylococcus aureus. Less common causes include fungi, parasitic and viral agents.

### Treatment and follow-up

The patient was treated with high dose oral prednisolone (1 mg/kg) with marginal improvement in symptoms; therefore, intravenous (IV) methylprednisolone was trialled as per British Society of Rheumatology guidelines.^7^ There was a significant improvement in the patient’s pain and inflammation following the administration of IV methylprednisolone. Blood results also reflected this improvement, with the CK level falling to 1190 U l^−1^. Subsequently, the patient was discharged with a reducing regime of steroids and pain relief after 7 days of inpatient admission. An outpatient follow-up with rheumatology was arranged. No follow-up MRI scan has been scheduled.

## Discussion

In early 2020, the World Health Organisation declared the coronavirus pandemic, with severe acute respiratory virus (SARS-CoV-2) identified as the causative agent for the coronavirus disease.^8^ Covid-19 is predominantly associated with respiratory tract pathology; however, it has also been found to affect the heart, kidneys, liver, pancreas and musculoskeletal systems.^9^ Whilst the disease is not fully understood, there is evidence to suggest how covid-19 can affect a multitude of body systems. This is due to the robust expression and distribution of angiotensin-converting enzyme 2 (ACE-2) in endothelial tissue of multiple organs including pulmonary, cardiac and skeletal muscle.^10^ SARS-CoV-2 mediates infection using the ACE-2 receptor as a host, suggesting that covid-19 can directly infect skeletal muscle causing the inflammatory response seen in myopathies such as covid-19-induced myositis.^10^ Thus, muscle weakness and myalgia are commonly reported symptoms of covid-19. Myositis differs from myalgia in that it has a more delayed onset, is more severe in pain intensity and is more focal in location.

Complications of myositis include rhabdomyolysis (RM), an uncommon but life-threatening condition reported in influenza virus, human immunodeficiency virus (HIV), hepatitis viruses type B and C and Covid-19.^11^ Rhabdomyolysis is a condition characterised by myoglobinuria and acute kidney disease.^11^ A study that investigated the prevalence and clinical features of RM in hospitalised covid-19 patients found a significant association between RM and worsening mortality.^11^ Therefore, early detection and treatment are imperative in reducing mortality in patients with covid-19. A similar case report has described an association between myositis, rhabdomyolysis and acute viral illness.^12^

Detailed history taking and physical examination are key to identifying the cause of myositis. Imaging can be used to support diagnosis with MRI being the preferred modality; however, the gold standard diagnostic test for myositis is muscle biopsy.

## Learning points

- Consider viral causes, including Covid-19, as a differential for patients presenting with suspected myositis.
- Diagnosis of inflammatory myopathy is suggested by clinical presentation, serological and radiological findings supported by skin and muscle biopsies.
- Diagnosis and treatment require a multispecialty approach including acute medical specialists, rheumatologists and radiologists.

## References

[1] Berth SH, Lloyd TE . Secondary causes of myositis. Curr Treat Options Neurol 2020; 22(11): 38. doi: 10.1007/s11940-020-00646-0 33041620 PMC7538050

[2] Leverenz D, Zaha O, Crofford LJ, Chung CP . Causes of creatine kinase levels greater than 1000 IU/L in patients referred to rheumatology. Clin Rheumatol 2016; 35: 1541–47. doi: 10.1007/s10067-016-3242-9 27041384 PMC4871697

[3] Lundberg IE, Miller FW, Tjärnlund A, Bottai M . Diagnosis and classification of idiopathic inflammatory myopathies. J Intern Med 2018; 176: 139–48. 10.1111/joim.12524 PMC5021058 27320359

[4] Ngan V, Sapsford S . Adult-onset dermatomyositis | DermNet. Internet. Available from: https://dermnetnz.org/topics/adult-onset-dermatomyositis (accessed 11 Apr 2023)

[5] Lundberg IE, Miller FW, Tjärnlund A, Bottai M . Diagnosis and classification of idiopathic inflammatory myopathies. J Intern Med 2016; 280: 39–51. doi: 10.1111/joim.12524 27320359 PMC5021058

[6] Ngan V, Menezes S, Health A, DermNet NZ . Livedo reticularis. Internet. 2016. Available from: https://dermnetnz.org/topics/livedo-reticularis (accessed 29 Jun 2022)

[7] Oldroyd AGS, Lilleker JB, Amin T, Aragon O, Bechman K, Cuthbert V, et al . Guidelines British Society for Rheumatology guideline on management of paediatric, adolescent and adult patients with idiopathic inflammatory myopathy. . 10.1093/rheumatology/keac115 10.1093/rheumatology/keac115 PMC9398208 35355064

[8] Adil MT, Rahman R, Whitelaw D, Jain V, Al-Taan O, Rashid F, et al . SARS-cov-2 and the pandemic of COVID-19. Postgrad Med J 2021; 1144: 110–16. Available from: https://pmj.bmj.com/content/97/1144/110 10.1136/postgradmedj-2020-138386 PMC10016996 32788312

[9] Iacobucci G . Long covid: Damage to multiple organs presents in young, low risk patients. BMJ. 2020. Available from: https://www.bmj.com/content/371/bmj.m4470 (accessed 1 Oct 2022)

[10] Ferrandi PJ, Alway SE, Mohamed JS . The interaction between SARS-cov-2 and ACE2 may have consequences for skeletal muscle viral susceptibility and myopathies. Journal of Applied Physiology 2020; 129: 864–67. doi: 10.1152/japplphysiol.00321.2020 32673162 PMC7832004

[11] Geng Y, Ma Q, Du Y-S, Peng N, Yang T, Zhang S-Y, et al . Rhabdomyolysis is associated with in-hospital mortality in patients with COVID-19. Shock 2021; 56: 360–67. doi: 10.1097/SHK.0000000000001725 33443364 PMC8354485

[12] Kietaibl AT, Fangmeyer-Binder M, Göndör G, Säemann M, Fasching P . Acute viral myositis: profound rhabdomyolysis without acute kidney injury. Wien Klin Wochenschr 2021; 133: 847–50. doi: 10.1007/s00508-021-01866-3 33905028 PMC8076669

